# Radiosensitivity of microscopic tumours of a transplantable mammary adenocarcinoma in mice.

**DOI:** 10.1038/bjc.1981.126

**Published:** 1981-06

**Authors:** J. Haveman, W. Jansen, E. van der Schueren, K. Breur

## Abstract

Evidence is presented that microscopic tumours (of a transplantable murine mammary carcinoma, M8013X) grow faster than larger, palpable, tumours. Microscopic tumours are also more radiosensitive than larger tumours. The decrease in radiosensitivity in larger tumours is prevented to a large extent by misonidazole, which has no significant effect on the radiosensitivity of microscopic tumours. The retardation in growth rate which occurs after the fast microscopic growth is probably related to the appearance of hypoxic cells. Both the decrease in growth rate and the progressive development of hypoxia may be caused by the relatively poorer blood flow in larger tumours. Part of the radioresistance in "large" tumours ( approximately 250 mm3) seems to be due to factors other than hypoxia; maybe cell-kinetic factors also play a role. The intrinsic radiosensitivity of tumour cells in microscopic tumours was assessed by means of a modified latency test: the Dq and Do were 2.2 and 2.5 Gy respectively. A number of factors which may influence the reliability of these estimates are discussed.


*Br. J. Cancer* (1981) **43**, 864

### RADIOSENSITIVITY OF MICROSCOPIC TUMOURS OF A TRANSPLANTABLE MAMMARY ADENOCARCINOMA IN MICE

J. HAVEMAN, W. JANSEN, E. VAN DER SCHUEREN AND K. BREUR

*From the Department of Radiotherapy, University of Amsterdam, The Netherlands*

Received 8 April 1980 Accepted 9 March 1981

**Summary.—Evidence is presented that microscopic tumours (of a transplantable murine mammary carcinoma, M8013X) grow faster than larger, palpable, tumours. Microscopic tumours are also more radiosensitive than larger tumours. The decrease in radiosensitivity in larger tumours is prevented to a large extent by misonidazole, which has no significant effect on the radiosensitivity of microscopic tumours. The retardation in growth rate which occurs after the fast microscopic growth is probably related to the appearance of hypoxic cells. Both the decrease in growth rate and the progressive development of hypoxia may be caused by the relatively poorer blood flow in larger tumours. Part of the radioresistance in "large" tumours ($\sim$250 mm$^3$) seems to be due to factors other than hypoxia; maybe cell-kinetic factors also play a role. The intrinsic radiosensitivity of tumour cells in microscopic tumours was assessed by means of a modified latency test: the $D_q$ and $D_o$ were 2·2 and 2·5 Gy respectively. A number of factors which may influence the reliability of these estimates are discussed.**

THE METABOLIC CONDITIONS and cell-kinetic characteristics of a tumour appear to change during its different growth phases. This has been suggested as having important repercussions on the effectiveness of anti-tumour agents such as ionizing radiation and hyperthermia. The effects of irradiation on a tumour would be influenced by the oxygenation status and the cell kinetics. Some evidence already exists that anoxic cells are absent from very small tumours and only appear during further growth (Suit *et al.*, 1960; Reinhold & de Bree, 1968; Shipley *et al.*, 1975, 1976; Fu *et al.*, 1976; Stanley *et al.*, 1978). It is very difficult to assess directly the influence of the cell kinetics of a tumour on its response to irradiation.

The irradiation of microscopic tumour locations is common practice in clinical radiotherapy, in what are usually called elective or adjuvant treatments (Fletcher, 1971, 1972; Newton & Spittle, 1969; Breur & Van der Schueren, 1979). The success of such types of treatments could be due to the relatively small number of cells to be inactivated, but would still be enhanced by a possible higher radiosensitivity of the tumour cells in early phases of growth.

In this study the radiosensitivity of a microscopic tumour is tested by means of a modified latency test (Clifton & Draper, 1963; Alfieri & Hahn, 1978). Moreover, the effects of the anoxic-cell sensitizer misonidazole (MISO) are studied. In this way information is obtained about the changes in radiosensitivity and the time of appearance of hypoxic cells during growth of a transplantable murine mammary carcinoma *in vivo*. Apart from the theoretical interest this would have practical implications, as it could provide guidelines on whether or not to test the possible usefulness of anoxic cell sensitizers for the radiotherapy of subclinical disease.

### MATERIALS AND METHODS

The M8013 tumour originated in 1950 as a mammary adenocarcinoma in a castrated oestrogen-stimulated male C57BL mouse at the NKI (Nederlands Kanker Instituut) (Van Dongen, 1961). The M8013X is a line derived from this tumour, which is kept by serial transplantation in male DBA2 × C57BL10 mice (obtained from the Centraal Proefdieren Bedrijf TNO, Zeist, Netherlands). The volume-doubling time of the M8013X tumour is 1·6–1·8 days. In most experiments described in this study, tumour implantation was performed by s.c. injection of small volumes, with a microlitre syringe, of a cell suspension in the right hind leg of 8–12-week-old mice. To obtain a cell suspension, an aseptically excised tumour was minced thoroughly with scissors in ~25 ml Eagle's minimum essential medium supplemented with 100 i.u./ml penicillin. The resulting cell suspension was separated from the tumour debris and the number of cells was counted, using lyssamine green exclusion as a test for viability. If necessary, the suspension was concentrated by centrifugation (8 min, 120 *g*) to a final concentration of $1–4 \times 10^6$ cells/ml. The cell yield was 2–4% (assuming $5 \times 10^8$ cells/g of tumour tissue; Steel, 1977). Very little clumping of cells was seen in the final suspension.

Tumour size was measured 3–5 times a week; tumour growth delay was determined comparing the time necessary for controls and treated animals to reach a tumour volume of 1 $cm^3$.

Irradiation was carried out by a Siemens Stabilipan apparatus operating at 250 kV and 14 mA, yielding a dose rate, after being filtered by 0·5mm Cu, of 1·89 Gy/min at the position of the tumour-bearing leg. Before irradiation the mice were anaesthesized with pentobarbitone sodium (Nembutal®, 50 mg/kg i.p.). The mice were kept at 30°C during anaesthesia and shielded with lead, except for the hind leg which protruded into the beam.

### RESULTS

Fig. 1 shows the effect of an irradiation (8 Gy) of tumours implanted on the leg. The tumour-bearing mice were irradiated on different days after implantation of $3 \times 10^4$ tumour cells into the right hind leg. About 10 days after s.c. injecting $3 \times 10^4$ cells, the tumours become palpable; 14 days after injection the tumour

FIG. 1.—The tumour growth delay caused by X-irradiation (8 Gy) on different days after implantation of $3 \times 10^4$ M8013X cells in the hind leg of mice (○) no MISO, (●) 0·5 mg/g MISO injected 30 min before irradiation. The numbers of animals per group were: irradiated 1 day after implantation, with or without MISO, 9; 4 days, 9; 7 days, 30; 10 days, 20; 12 days, 9; 14 days, 9; 13 days with MISO, 9; 13 days without MISO, 5. The error bars represent s.e.

volume is ~250 $mm^3$. A significant drop in radiosensitivity is found after about 10 days. The decrease in radiosensitivity is prevented largely, but not completely, by MISO.

Tumour growth delay caused by irradiation can be correlated with the relative survival of tumour cells, using a "titration" curve as in Fig. 2 (Alfieri & Hahn, 1978; Haveman *et al.*, 1980). In this way the "intrinsic" radiosensitivity of the (microscopic) tumours can be assessed. In a titration curve it is shown that implanting lower cell numbers leads to an increase in tumour growth delay. Irradiation of a tumour leads to a reduction in cell number and the extra time which is required by a tumour to grow to a certain volume after irradiation can be used to estimate this reduction in cell number, by matching it, on a titration curve, with the extra tumour growth time due to implantation of a certain lower cell number. In this way survival curves can be obtained, as

FIG. 2.—Dependence of the tumour growth time to a volume of 1 $cm^3$ on the number of dye-excluding M8013 cells injected in two different locations: (○) s.c. in the hind leg and (●) s.c. on the back. The numbers of animals per group were: leg 10; back 20, with the exception of the group receiving $3 \times 10^5$ cells (10). In brackets the number of "takes" per group when not 100%. The error bars represent s.e.

shown in Fig. 3. The titration curves in Fig. 2 show also that:

(1) When lower cell numbers are implanted, some of the injected mice failed to develop a tumour ("no take") and when "no takes" occur the titration curve deviates from the straight line which can be drawn through the data points with 100% takes.

(2) The tumour growth time to a certain volume not only depends on the number of cells implanted, but also on the site of the implantation.

(3) From the slope of the curve of tumour growth time against the logarithm of the number of implanted cells, a doubling time of 2·0 days is obtained. This doubling time does not differ significantly for the different sites of implantation. From the measurements of tumour volume during exponential growth on the leg or the back, a doubling time of 1·6–1·8 days is obtained, which is slightly shorter than the value from a "titration" curve. Owing to the occurrence of "no takes" at lower cell numbers in a titration curve, the dose range in the survival curves (Fig. 3) which can be studied is limited. Moreover, the deviation from the straight line when lower cell numbers are implanted has to be taken into account. Titration curves were repeated for each independent experiment shown in Fig. 3 (10 animals per treatment group).

The tumour growth time to 1 $cm^3$ varies slightly between experiments; starting from $10^5$ cells injected s.c. into the leg, the mean value is (19 independent experiments, 10 animals each) 14·4 days, standard deviation 1·7 days.

When M8013X cells were "titrated" on the leg of pre-irradiated mice (5 Gy total body 3 days before implantation) the tumour growth times and the slope of the resulting curve did not differ significantly from a titration curve on animals without treatment; "no takes" however appeared only at lower numbers of cells injected.

As shown in Fig. 3, no significant differences can be found in radiation sensitivity, whether the cells were irradiated before implantation or 4 h, 3 days and 10 days after implantation of $3 \times 10^4$ cells. The same curve fits the data of Fig. 3(a) and (b). The data in Fig. 3 confirm those from the independent experiment shown in Fig. 1. The decrease in radiosensitivity at Day 10 in Fig. 1 is not, however, as clear in Fig. 3(b); this may be due to the spread in the data, or to slight variations in growth time to a certain tumour volume, starting with $3 \times 10^4$ cells in independent experiments.

Dose–response curves, with and without MISO, for tumours with a volume ~250 $mm^3$ are shown in Fig. 4(a). In Fig. 4(b) the data of Fig. 4(a) are redrawn so that they may be compared with the

FIG. 3.—The assumed relative survival of M8013X cells after X-irradiation. (a) (○) irradiated before implantation, (●) irradiated 4 h after implantation of $3 \times 10^4$ cells in the hind leg. Treatment groups contained 8–10 animals. (b) irradiated 4 h (○) (▼), 3 days (△), or 10 days (●) after implantation of $3 \times 10^4$ cells in the hind leg; (▼) with MISO. The treatment groups consisted of 5–8 animals. TGD: tumour growth delay. The assumed relative survival is obtained by matching TGD on a titration curve on the leg as described in the text.

data for the microscopic tumours. The large tumours appear to be much more radioresistant than microscopic tumours. MISO enhances the radiosensitivity of the 250mm³ tumours, even at low doses, but not completely to the level of the microscopic tumours. The $D_q$ and $D_0$ values for microscopic tumours (Fig. 4(b)) are 2·2 and 2·5 Gy respectively, and for 250-mm³ tumours without MISO 4·5 and 7·0 Gy, and with MISO 2·8 and 3·7 Gy respectively.

### DISCUSSION

Microscopic tumours of M8013X appear to be significantly more radiosensitive than the (macroscopic) palpable tumours. The relatively low radiosensitivity of the large tumours can be enhanced considerably by MISO. Very probably the radioresistance of the large tumours is thus at least in part the result of the progressive development of hypoxia in the tumour during growth. Large tumours, even in the presence of MISO, are more radioresistant than microscopic tumours (Fig. 4(b)). This may be due to cell-kinetic effects; as the fraction of actively proliferating cells could be decreased in these large tumours. The dose–response curve (Fig. 4) for 250mm³ tumours shows that these are more radioresistant than microscopic tumours, even at low doses. The effect of the hypoxic cell sensitizer is also evident at lower doses. This is very probably because the hypoxic cell fraction in these 250mm³ tumours is very large. In the mean time, we found that effects due to hypoxia in larger tumours were further enhanced by the Nembutal anaesthesia (Wondergem *et al.*, 1981). Similar effects of Nembutal (for other tumours) have been described by Denekamp *et al.* (1979). The progressive development of hypoxia during growth (Fig. 1) is presumably due

Fig. 4.—(a) The tumour growth delay caused by X-irradiation of palpable M8013X tumours ($\sim$250 mm$^3$) located on the hind leg of mice: (○) without MISO, (●) 0·5 mg/g MISO injected i.p. 30 min before irradiation. 9–10 animals per treatment group. In (b) the data are replotted in such a way that tumour growth delay in palpable tumours can be compared with the assumed relative survival of microscopic tumours. The dashed curve is redrawn from Fig. 3.

to the relatively insufficient vascularization of the tumour, compared to the tumour growth rate. This apparently becomes evident at a certain tumour volume. (When in our experiments the tumours start to be palpable, tumour volume $< 50$ mm$^3$.)

The fact that the growth time to a certain tumour volume is dependent on the site of implantation may be caused by differences in lag time before cells start to grow at the different sites, or by differences in take efficiency of the cells. A certain fraction of cells may be lost upon implantation, depending on the site of implantation. In the experiment shown in Fig. 2 the tumour growth time to 1 cm$^3$ on the leg and the back differs by 4·5 days (*i.e.* 2·2–2·8 doubling times) which may mean that the take efficiency on the back is 15–20% of that on the leg.

It is important to consider differences in tumour growth time at different sites. For our experiments on the radiosensitivity of microscopic tumours, implantation of tumour cells was done as far as possible on the same site on the lower leg, using a small inoculum (25 $\mu$l).

In Fig. 3 the intrinsic radiosensitivity of tumour cells in microscopic tumours is assessed. A number of factors may limit the reliability of an estimation of the radiosensitivity in this way, the method described may give a fair estimate for microscopic tumours, but possibly not for larger palpable tumours.

(1) The slightly longer doubling time obtained from a "titration" curve (Fig. 2) may lead to a slight underestimation of the radiosensitivity, as a doubling time of 1·7 days may be taken as more realistic.

(2) Irradiation of tumours may lead to fast regrowth (Van Peperzeel, 1970), an effect which is difficult to evaluate in this model; again, a false estimate of the radiosensitivity using the "titration" curve may be the result. However, accelerated regrowth after irradiation probably does not play an important role in microscopic tumours, which must be supposed to grow already relatively fast (see below).

(3) The error due to cell-kinetic effects during microscopic growth is apparently not very large; there are no significant changes in radiosensitivity during the first 7 days after inoculation.

(4) Effects due to irradiation of the tumour-bed, in the dose range studied, are probably not very large; there is no significant difference in apparent relative survival of cells irradiated before or after implantation (Fig. 3) nor are there any significant differences in tumour growth delay during the first 7 days after inoculation (Fig. 1).

(5) Cell loss, which may occur in large tumours, is probably negligible in microscopic tumours.

"Large" ($250 mm^3$) tumours (Fig. 4a) are more radioresistant than the microscopic tumours even at low doses, and there is no clear break in the dose–response curve (Jansen, 1980; Wondergem *et al.*, 1981); possibly the hypoxic cell fraction in these tumours is at least 50%.

It has to be postulated that the doubling time during microscopic growth is much shorter than during macroscopic growth. Even when it is assumed that all cells injected contribute to growth, the tumour growth time to 1 $cm^3$ on the leg (1 $cm^3$ is assumed to contain $\sim 5 \times 10^8$ malignant cells) is very short, and cannot be explained by a volume-doubling time of 1·6–1·8 days. A volume of 50 $mm^3$ after implantation of $10^5$ cells on the leg is reached in 7–8 days. From 50 $mm^3$ to 1 $cm^3$ (4·3 doublings) takes 7·0 days, which means a doubling time of 1·7 days. For the first 8 doublings to reach 50 $mm^3$, each doubling must have occurred in a maximum of 24 h. Cell lines derived from the tumour, growing *in vitro*, had doubling times of about 13 h, and a cell-cycle time of 14·5 h has been described for the M8013 tumour (Van Peperzeel, 1970). From the slope of the curve of tumour growth time against the logarithm of the number of implanted cells (Fig. 2), a doubling time of 2·0 days is obtained. The fact that the doubling time from the slope of the titration curve is in excess of 1·7 days probably means that microscopic exponential growth when a smaller number of cells is inoculated is dominated only for a limited time by the fast growth, a relatively large part of the microscopic growth already occurring with the doubling time of macroscopic growth, when a smaller number of cells is inoculated. Another possibility is that lower cell numbers lead to a longer lag phase before cell proliferation starts.

The retardation of growth rate which occurs after the fast microscopic growth is probably related to the appearance of a hypoxic cell fraction, as assessed in Fig. 1.

In conclusion, it can be said that our results further confirm that small tumours are more radiosensitive than larger ones. In larger tumours (with $> 3 \times 10^7$ cells in this model) the radioresistance may be explained at least in part by the presence of hypoxic cells, as the radioresistance can be largely removed by MISO. Other, such as cell-kinetic, factors are not excluded as large tumours, even in the presence of hypoxic-cell sensitizer, are more radioresistant than microscopic tumours. Microscopic tumours grow faster than larger tumours, the retardation in growth rate occurring after the fast microscopic growth being very probably related to the appearance of hypoxic cells, probably caused by a relatively poor blood flow in larger tumours. The method of relating implanted cell numbers with tumour growth time can be used to obtain an estimate of the intrinsic radiosensitivity of tumour cells *in vivo* over a limited dose range. Fraction sizes normal to clinical practice are well within the dose range studied. However, one should be aware of a number of factors which may limit the direct applicability of the method to larger palpable tumours.

We are indebted to Mrs F. Rijnders, A. Braun and N. Middelburg and to Mr P. Zum Vörde Sive Vörding for their skilful technical assistance.

This work was supported in part by a grant from the KWF (Koningin Wilhelmina Fonds).

### REFERENCES

ALFIERI, A. A. & HAHN, E. W. (1978) An *in situ* method for estimating cell survival in a solid tumor. *Cancer Res.*, **38**, 3006.

BREUR, K. & VAN DER SCHUEREN, E. (1979) Adjuvant therapy in the management of osteosarcoma: Need for critical reassessment. *Recent Results Cancer Res.*, **68**, 5.

CLIFTON, K. H. & DRAPER, N. R. (1963) Survival-curves of solid transplantable tumour cells irradiated *in vivo*: A method of determination and statistical evaluation; comparison of cell-survival and $^{32}$P-uptake into DNA. *Int. J. Radiat. Biol.*, **7**, 515.

DENEKAMP, J., TERRY, N. H. A., SHELDON, P. W. & CHU, A. M. (1979) The effect of pentobarbital anaesthesia on the radiosensitivity of four mouse tumours. *Int. J. Radiat. Biol.*, **35**, 277.

FLETCHER, G. H. (1971) Control by irradiation of peripheral lymphatic disease in breast cancer. *Am. J. Roentgenol.*, **111**, 115.

FLETCHER, G. H. (1972) Elective irradiation of subclinical disease in cancers of the head and neck. *Cancer*, **29**, 1450.

FU, K. K., PHILLIPS, T. L. & WHARAM, M. D. (1976) Radiation response of artificial pulmonary metastases of the EMT-6 tumor. *Int. J. Radiat. Oncol. Biol. Phys.*, **1**, 257.

HAVEMAN, J., VAN DER SCHUEREN, E. & BREUR, K. (1980) Possibilities of an *in vivo* cell titration method for the assessment of the radiosensitivity of microscopic tumours. *Br. J. Cancer*, **41** (Suppl. IV), 304.

JANSEN, W. (1980) *Combination of hyperthermia and radiation in the treatment of experimental tumours in mice.* Thesis, University of Amsterdam.

NEWTON, K. A. & SPITTLE, M. F. (1969) An analysis of 40 cases treated by total thoracic irradiation. *Clin. Radiol.*, **20**, 19.

REINHOLD, H. S. & DE BREE, C. (1968) Tumour cure rate and cell survival of a transplantable rat rhabdomyosarcoma following X-irradiation. *Eur. J. Cancer*, **4**, 367.

SHIPLEY, W. U., STANLEY, J. A. & STEEL, G. G. (1975) Tumor size dependency in the radiation response of the Lewis lung carcinoma. *Cancer Res.*, **25**, 2488.

SHIPLEY, W. U., STANLEY, J. A. & STEEL, G. G. (1976) Enhanced tumor cell radiosensitivity in artificial pulmonary metastases of the Lewis lung carcinoma. *Int. J. Radiat. Oncol. Biol. Phys.*, **1**, 261.

STANLEY, J. A., PECKHAM, M. J. & STEEL, G. G. (1978) Influence of tumour size on radiosensitization by misonidazole. *Br. J. Cancer*, **37**, (Suppl III), 220.

STEEL, G. G. (1977) *Growth kinetics of tumours.* Oxford: Clarendon Press.

SUIT, H. D., SCHLACHTER, L. & ANDREWS, J. R. (1960) "Oxygen effect" and tumor size as related to response of C3H/Ba adenocarcinoma to local X-irradiation. *J. Natl Cancer Inst.*, **24**, 1271.

VAN DONGEN, J. A. (1961) Haematogene metastasen. Experimenteel onderzoek en literatuuroverzicht over de factoren, die het ontstaan, de localisatie en de uitgroei van tumormetastasen beïnvloeden. *Scheltema en Holkema*, Amsterdam.

VAN PEPERZEEL, H. A. (1970) *Patterns of tumor growth after irradiation.* Thesis, University of Amsterdam.

WONDERGEM, J., HAVEMAN, J., VAN DER SCHUEREN, E., VAN DEN HOEVEN, H. & BREUR, K. (1981) The influence of misonidazole on the radiation response of murine tumors of different size: Possible artifacts caused by pentobarbital sodium anesthesia. *Int. J. Radiat. Oncol. Biol. Phys.*, (in press).


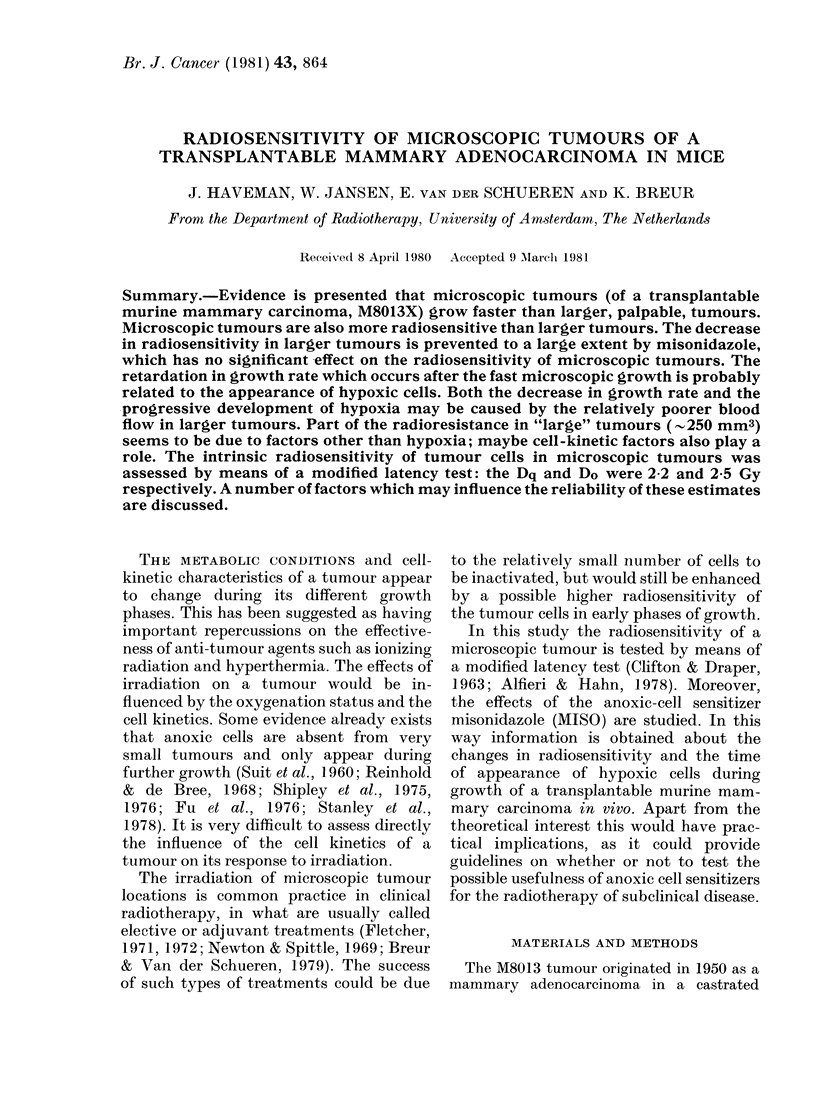

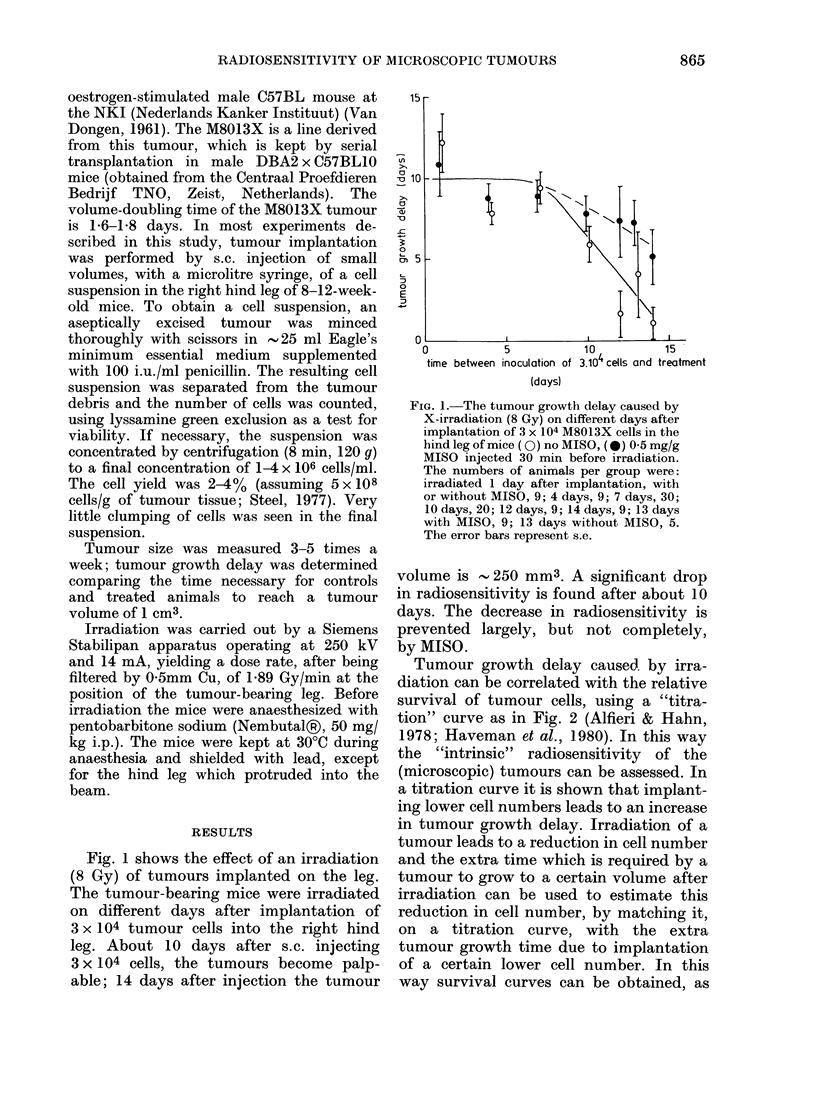

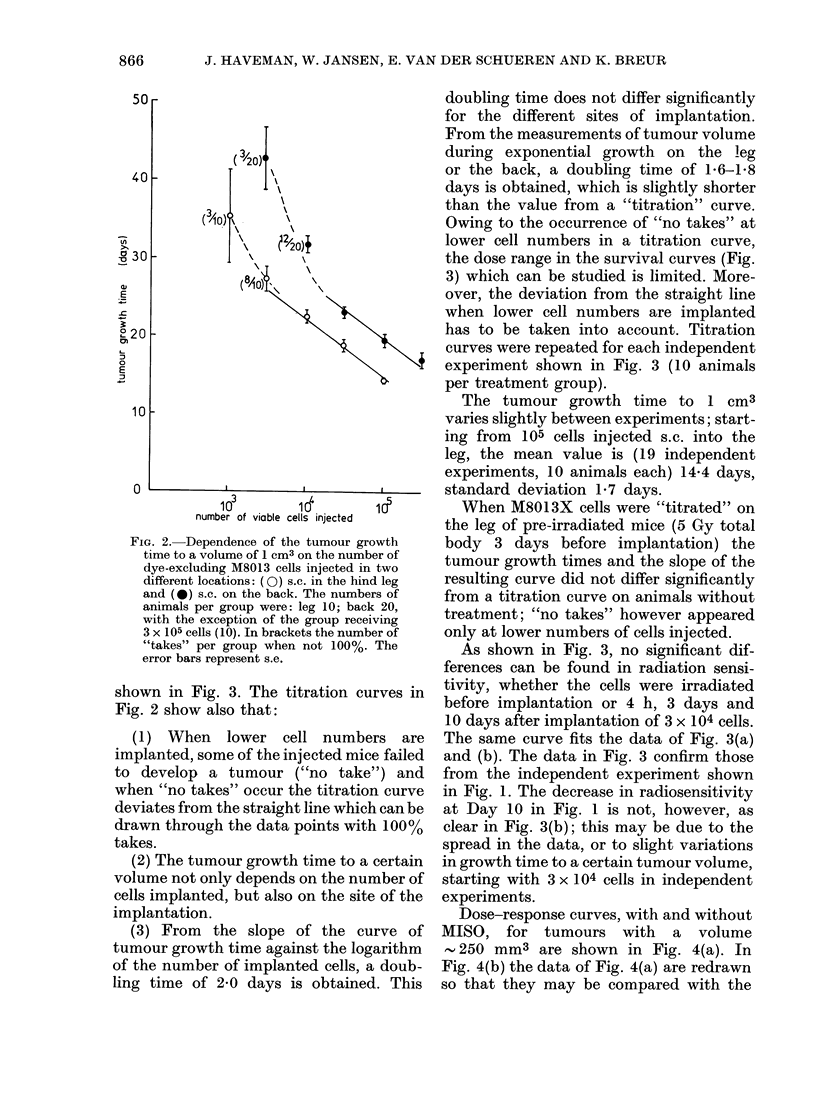

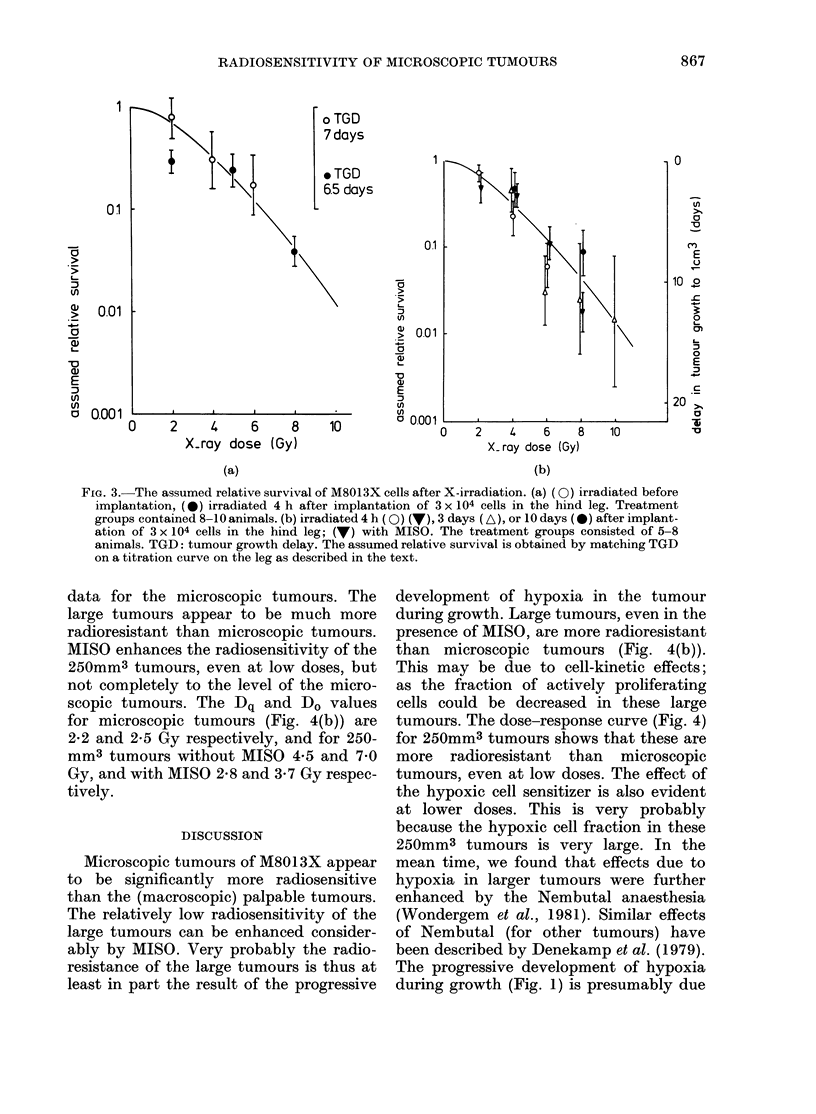

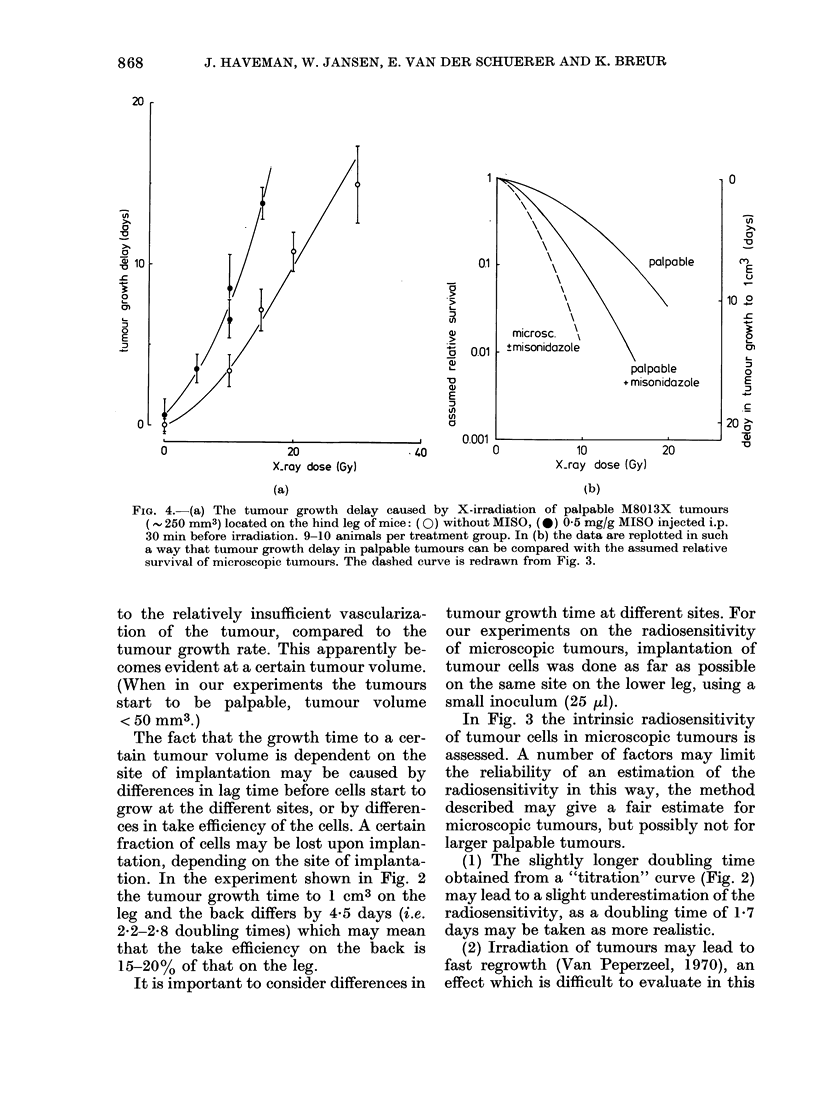

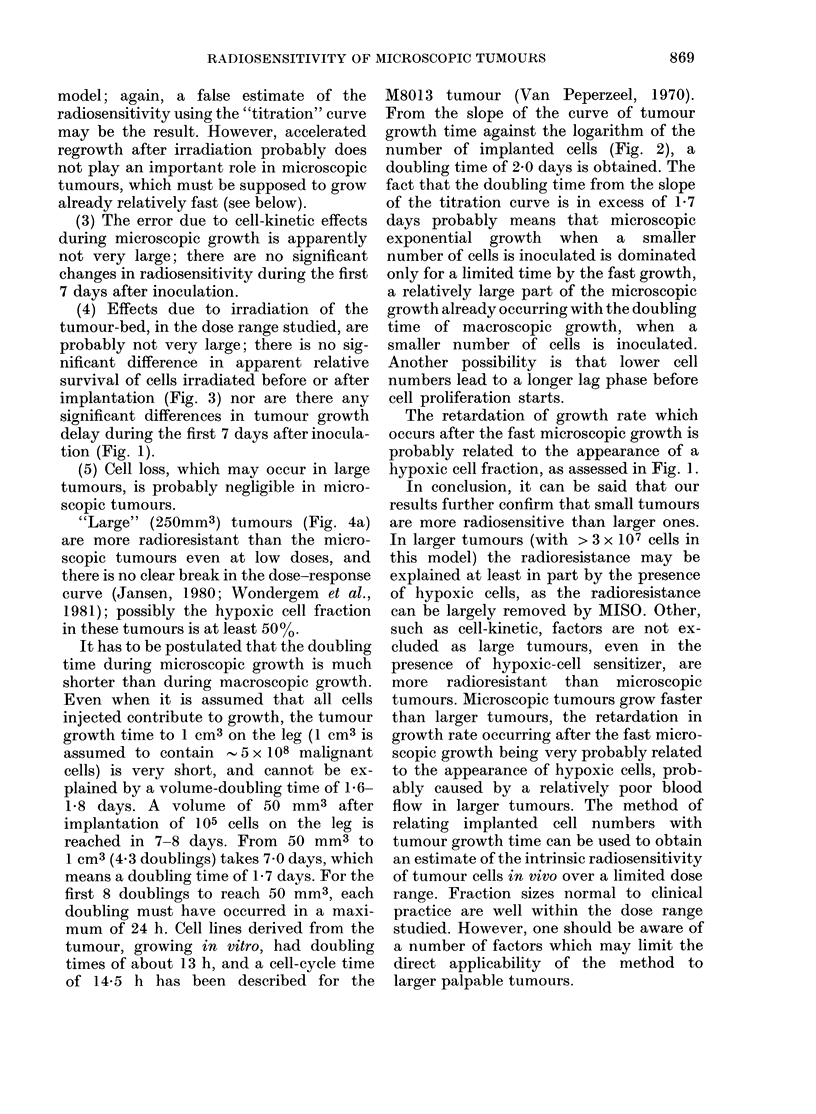

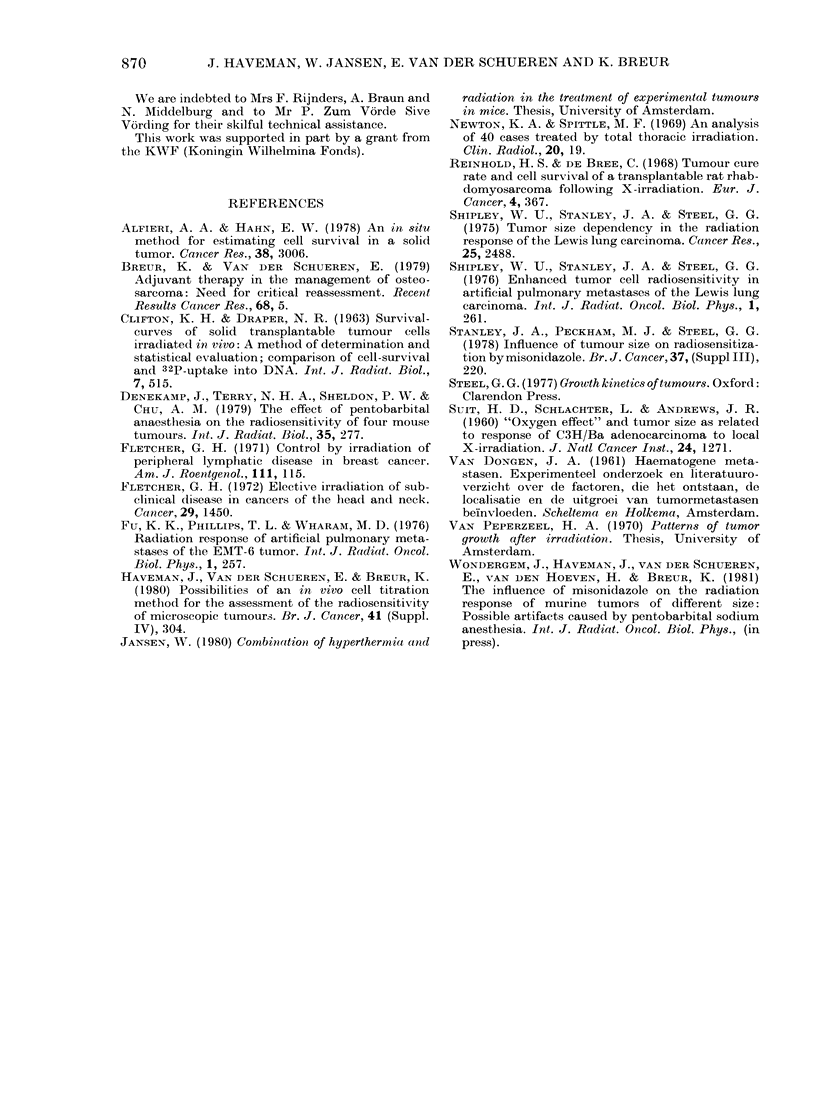


## References

[OCR_00731] Alfieri A. A., Hahn E. W. (1978). An in situ method for estimating cell survival in a solid tumor.. Cancer Res.

[OCR_00736] Breur K., van der Schueren E. (1978). Adjuvant therapy in the management of osteosarcoma: need for critical reassessment.. Recent Results Cancer Res.

[OCR_00742] CLIFTON K. H., DRAPER N. R. (1963). SURVIVAL-CURVES OF SOLID TRANSPLANTABLE TUMOUR CELLS IRRADIATED IN VIVO: A METHOD OF DETERMINATION AND STATISTICAL EVALUATION; COMPARISON OF CELL-SURVIVAL AND 32-P-UPTAKE INTO DNA.. Int J Radiat Biol Relat Stud Phys Chem Med.

[OCR_00750] Denekamp J., Terry N. H., Sheldon P. W., Chu A. M. (1979). The effect of pentobarbital anaesthesia on the radiosensitivity of four mouse tumours.. Int J Radiat Biol Relat Stud Phys Chem Med.

[OCR_00756] Fletcher G. H. (1971). Control by irradiation of peripheral lymphatic disease in breast cancer.. Am J Roentgenol Radium Ther Nucl Med.

[OCR_00761] Fletcher G. H. (1972). Elective irradiation of subclinical disease in cancers of the head and neck.. Cancer.

[OCR_00766] Fu K. K., Phillips T. L., Wharam M. D. (1976). Radiation response of artificial pulmonary metastases of the EMT6 tumor.. Int J Radiat Oncol Biol Phys.

[OCR_00772] Haveman J., Van Der Schueren E., Breur K. (1980). Possibilities of an in vivo cell titration method for the assessment of the radiosensitivity of microscopic tumours.. Br J Cancer Suppl.

[OCR_00784] Newton K. A., Spittle M. F. (1969). An analysis of 40 cases treated by total thoracic irradiation.. Clin Radiol.

[OCR_00789] Reinhold H. S., De Bree C. (1968). Tumour cure rate and cell survival of a transplantable rat rhabdomyosarcoma following x-irradiation.. Eur J Cancer.

[OCR_00818] SUIT H., SCHLACHTER L., ANDREWS J. R. (1960). "Oxygen effect" and tumor size as related to response of C3H/Ba adenocarcinoma to local X irradiation.. J Natl Cancer Inst.

[OCR_00801] Shipley W. U., Stanley J. A., Steel G. G. (1976). Enhanced tumor cell radiosensitivity in artificial pulmonary metastases of the Lewis lung carcinoma.. Int J Radiat Oncol Biol Phys.

[OCR_00795] Shipley W. U., Stanley J. A., Steel G. G. (1975). Tumor size dependency in the radiation response of the Lewis lung carcinoma.. Cancer Res.

[OCR_00808] Stanley J. A., Peckham M. J., Steel G. G. (1978). Influence of tumour size on radiosensitization by misonidazole.. Br J Cancer Suppl.

